# COPS: Detecting Co-Occurrence and Spatial Arrangement of Transcription Factor Binding Motifs in Genome-Wide Datasets

**DOI:** 10.1371/journal.pone.0052055

**Published:** 2012-12-18

**Authors:** Nati Ha, Maria Polychronidou, Ingrid Lohmann

**Affiliations:** Centre for Organismal Studies (COS) Heidelberg, University of Heidelberg, Heidelberg and CellNetworks – Cluster of Excellence Germany, Heidelberg, Germany; Université de Nantes, France

## Abstract

In multi-cellular organisms, spatiotemporal activity of *cis*-regulatory DNA elements depends on their occupancy by different transcription factors (TFs). In recent years, genome-wide ChIP-on-Chip, ChIP-Seq and DamID assays have been extensively used to unravel the combinatorial interaction of TFs with *cis*-regulatory modules (CRMs) in the genome. Even though genome-wide binding profiles are increasingly becoming available for different TFs, single TF binding profiles are in most cases not sufficient for dissecting complex regulatory networks. Thus, potent computational tools detecting statistically significant and biologically relevant TF-motif co-occurrences in genome-wide datasets are essential for analyzing context-dependent transcriptional regulation. We have developed COPS (Co-Occurrence Pattern Search), a new bioinformatics tool based on a combination of association rules and Markov chain models, which detects co-occurring TF binding sites (BSs) on genomic regions of interest. COPS scans DNA sequences for frequent motif patterns using a Frequent-Pattern tree based data mining approach, which allows efficient performance of the software with respect to both data structure and implementation speed, in particular when mining large datasets. Since transcriptional gene regulation very often relies on the formation of regulatory protein complexes mediated by closely adjoining TF binding sites on CRMs, COPS additionally detects preferred short distance between co-occurring TF motifs. The performance of our software with respect to biological significance was evaluated using three published datasets containing genomic regions that are independently bound by several TFs involved in a defined biological process. In sum, COPS is a fast, efficient and user-friendly tool mining statistically and biologically significant TFBS co-occurrences and therefore allows the identification of TFs that combinatorially regulate gene expression.

## Introduction

Cell-type specific gene expression results from the combinatorial interaction of transcription factors (TFs) with *cis*-regulatory DNA elements, which are instructed by clusters of TF binding sites (TFBSs) [1], [2]. Notably, not only the presence of TFBSs but also their spatial arrangements within *cis*-regulatory modules (CRMs) is a critical aspect of spatiotemporal regulation of gene expression. Preferred TFBS spacing may indicate formation of regulatory protein complexes mediated by closely adjoining TFBSs (Δbp ≈ 10 bp) [3], indirect interactions mediated by adaptor proteins (Δbp = multiples of 10 bp) and direct/indirect interactions of distant TFs mediated by chromatin structures, i.e. chromatin loops (Δbp = multiples of 100 bp) [4], [5].

In recent years, the wide-spread use of genome-wide chromatin-profiling methods such as chromatin immunoprecipitation (ChIP) followed by microarray analysis (ChIP-on-Chip) or massively parallel sequencing (ChIP-Seq) and DNA-adenine methyltransferase identification (DamID) has generated the *in vivo* binding maps of numerous TFs. In several cases, the determination of *in vivo* binding patterns for a set of TFs involved in the same biological process has allowed the dissection of combinatorial TF interactions at the genome-wide level. Prominent examples include the elucidation of the transcriptional networks controlling muscle and nervous system development in *Drosophila* [6], [7] as well as the identification of TF combinations instructing heart development in mammals [8].

Despite the increasing availability of genome-wide DNA interaction data for a vast number of TFs, the acquisition of binding profiles for all TFs essential for the regulation of cell- or tissue-type specific developmental processes is so far almost impossible in higher organisms. Furthermore, TFs are often expressed in many different cell types and yet manage to co-ordinate cell-type specific transcriptional programs via their collaboration with different co-regulators. Therefore, as single or few TF binding profiles are not sufficient for dissecting complex regulatory networks, computational programs mining statistically significant and biologically relevant co-occurrences of TF motifs are crucial for the identification of co-regulatory TFs. Computational tools that scan pre-selected sequences or whole genomes for homotypic or heterotypic clusters of TFBSs are the most effective among the existing approaches implemented for the identification of combinatorially regulated CRMs (reviewed in [9]).

In this study, we present COPS (Co-Occurrence Pattern Search), a computational tool mining frequent co-occurrences of TF motifs in genome-wide data using a combination of association rules and Markov chain models. In addition to detecting motif co-occurrences, COPS reports the preferred spatial arrangement of the TFBSs, an important feature of cell-type specific CRM activities. The performance of COPS was evaluated by analyzing cell type-specific *in vivo* binding regions for two *Drosophila* and one mouse genome-wide datasets [6], [7], [8]. In all three cases, COPS retrieved the BSs of known co-regulators of the analyzed TFs, demonstrating that it is a powerful tool for detecting biologically relevant TFBS co-occurrences and is thus useful for identifying novel transcriptional co-regulators. The availability of *in vivo* binding data for known co-regulatory TFs allowed us to validate the *in vivo* significance of the detected motif co-occurrences. Importantly, in all cases COPS calculated a substantial number of co-occurring motifs for TFs with known functions in the selected tissue-specific biological processes, suggesting their combinatorial activity on selected CRMs. In comparison to existing computational approaches, COPS is more suitable for handling large datasets combined with extensive motif collections. Furthermore, it detects preferred spatial arrangements of BSs, an important aspect of regulatory TF complex formation. In sum, we show that COPS is a powerful, time-efficient and user-friendly bioinformatics tool for identifying co-regulatory TFs that control cell- and tissue-specific target gene expression in a combinatorial manner.

## Materials and Methods

### Datasets Analyzed Using COPS

#### Drosophila mesoderm dataset

The genomic regions bound by Twist (Twi), Myocyte enhancer factor 2 (Mef2), Tinman (Tin), Bagpipe (Bap) and Biniou (Bin) at the embryonic stages 10 to 11 (6–8 h of development) [6] were analyzed. Due to the large variation in the length of ChIP-on-Chip identified regions we analyzed regions of a length ≤1000 bp (809 Twi-bound, 934 Mef2-Bound, 666 Bin-bound, 407 Bap-bound and 514 Tin-bound sequences).

#### Drosophila neural stem cell TF dataset

Genomic regions bound by the neurogenic factors Prospero (Pros) (2611 sequences), Asense (Ase) (2745 sequences), Deadpan (1426 sequences) and Snail (4000 sequences) at the embryonic stages 10 to 11 were defined by extending the DamID-identified regions [7] by 500 bp on each end.

#### Mouse cardiac TF dataset

We analyzed the genomic regions bound by Mef2a (883 sequences), GATA-binding protein 4 (GATA4) (473 sequences), NK2 TF related, locus 5 (Nkx2.5) (386 sequences) and Serum response factor (Srf) (1291 sequences), as defined by ChIP-on-Chip in HL-1 cells [8].

### Motifs Used for Scanning the Datasets

All TF binding motifs annotated in open source databases (JASPAR [10], TRANSFAC [11]) were used. Additionally, the optimized Position Weight Matrices (PWMs) for Twi, Mef2, Tin, Bap and Bin [6] were used for detecting BSs of these TFs in both *Drosophila* datasets. The Pros PWM described by Down et al. (2007) [12] was used for detecting Pros BSs in both *Drosophila* datasets. The PWM of GATA1 (JASPAR, TRANSFAC) was used for detecting GATA4 BSs, as motifs recovered from GATA4 bound regions were reported to match the GATA1 motif [13]. All motifs used for scanning for BSs of the main TFs from all three datasets are shown in Figure S1.

### Analysis of the Tissue-specific Properties of the Co-occurring TFs

Information concerning the tissue-specific expression and developmental functions of *Drosophila* and mouse TFs was retrieved from FlyBase [14] and UniProtKB [15], [16] respectively.

### Sequence Overlap Analysis

In order to compare the *in vivo* overlap with the expected (background) overlap, an overlap analysis was performed for the frequent motif patterns for which genome-wide data was available. The expected overlap was measured by randomly permuting (1000 times) the same number of regions bound by one TF through the genome and the mean overlap was subsequently calculated. The significance of the observed compared to the expected overlap was calculated by assuming that the overlap follows a Poisson distribution.

### COPS Implementation

#### Scanning the sequences for TF motifs

Motif position frequency matrices (PFMs) describing the probability of nucleotide distribution at each position were used for detecting TFBSs. First a weight sum of log scaled score was calculated as described in [17]. Since the scores vary for the different motifs, the score distribution of each motif was calculated as described in [18], in order to estimate the threshold cut-off for a given p-value.

#### Frequent pattern (FP)-tree building

A data mining method based on a frequent pattern (FP) growth algorithm was used. This method uses an extended prefix tree structure for storing quantitative information about frequent patterns. The FP-tree is composed of a header table and nodes representing items, and each node has three member variables node-name, node-count and node-link. The node-name represents the item name, the node-count records the path reaching this node and the node-link links to the next node in the FP-tree carrying the same node-name. The header table consists of the item name and head of node-link, which points to the first node in the FP-tree carrying the item name. The nodes were arranged in a way that frequent co-occurrence nodes would have higher chance for sharing nodes [19].

Let 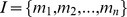 be a set of motifs, and a database 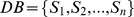 is the motif occurrence in 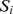 (each sequence) which contain a set of motifs in 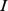. The support of a pattern 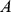, which is a set of motifs, is the number of sequences containing 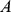 in 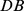. The set of motifs 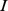 were sorted alphabetically, the ordering is defined as 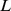. The order of the motifs is important, as each path of the tree will follow this order.

The FP-tree is gradually built by incorporating results from scanning each sequence. A list of motifs found on the sequence will be first sorted according to 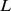, and then sequentially inserted into the tree. The insertion starts from the root node and recursively traverses the tree to update node count and node link until it has reached the last node. The major operations involved in the update process are node count increment and new node creation. Each sequence 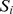 in the 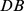 is mapped to one path in the FP-tree, and the occurrence of each motif in the sequence is stored in the FP-tree.

The header table together with the constructed FP-tree were used for mining frequent patterns. Starting from the node-link of each node-name in the header table, the frequent pattern is generated by concatenating the nodes in the same prefix path. Their corresponding number of occurrence is calculated by nodes accumulation and prefix path count adjustment.

#### Statistical validation of frequent patterns

In our approach, a log likelihood score based on a Markov model was used to calculate the statistical significance of the frequent patterns. 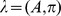 denotes a Markov model, where 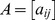 is the state transition probability matrix, and each transition probability is defined as 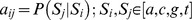. The probability of the observed sequence 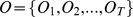, considering the Markov model 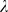 can be calculated as: 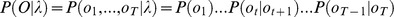. In our approach we trained two third-order Markov models 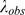 and 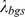 using the observed sequences (genome-wide dataset) and background sequences (all non-coding sequences). The probability of observing a sequence “atgta” using a third-order Markov model is calculated as: 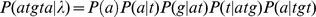.

Assuming both Markov models are ergodic, the empirical Kullback-Leibler divergence (KLD) between two Markov models is
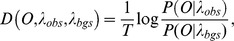
where 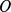 is a sequence of observation and 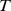 is the length of the observed sequence. 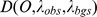 was estimated using Monte Carlo simulations [20], and it can be explained as how well the 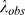 scores the observation sequence relative to the 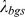. A score based on the KLD for the frequent motif pattern is defined as:



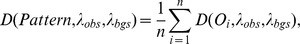
where 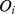 is the BS of the frequent motif pattern in each sequence, and 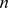 is the total number of BSs of the frequent motif pattern. For instance, for a pattern composed of motifs A and B, the subset of sequences containing motifs A and B is used to calculate the score. 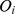 is the BS of A and B in each sequence, and 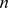 is the total number of BSs of motif A and B. In our analysis, we used genome-wide data sets generated for a given TF (main TF), therefore any pattern containing this TF will have a high score, as the motifs of this TF are enriched in this dataset. In order to reduce the bias caused by the main TF, the main TF binding sites are not included in the calculation. The background was estimated by randomly taking a subset of DNA sequences from the observed sequences and calculating 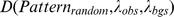. Assuming the score follows a normal distribution, we define a Z score as:



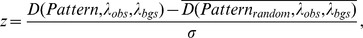
where 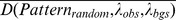 is the estimated mean score of the random pattern, and 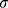 is the estimated standard deviation of the random pattern.

#### Analysis of preferred distance arrangements between co-occurring TFBSs

A distance analysis was carried out in order to detect distance preferences between motif pairs in the frequent motif patterns. The motifs were scanned on the sample sequences and their corresponding coordinates were used to calculate the pair-wise distance. The pair-wise distance is always representing the distance between the last nucleotide of the first motif and the first nucleotide of the second motif of the pair. The calculated distances between the motifs in the pair were distributed in equally spaced intervals of 10 bp. The background was estimated by analyzing the pair-wise distance of the motifs in the pair in randomly selected sequences from non-coding regions in the genome. The significance of the interval was calculated by fitting the interval data to a binomial distribution and the p-value was calculated for each interval.

### GO Term Analysis of Genes Associated to Genomic Regions Containing Closely Spaced TFBSs

The non-coding regions upstream and downstream of every gene in the *Drosophila* genome were scanned using a 500 base pair window, in order to detect short distance motif pairs (Bin/Tin: −1–9 bp, Mad/Twi: −1–9 bp and Ase/Dpn: −1–9 bp). The GO terms of the genes associated with regions containing the respective short distance pairs were compared to the rest of the genes in the genome using the Fisher exact test.

### Comparison to Other Computational Approaches

The relative performance of COPS was compared to ModuleDigger [21] and CPModule [22] using the Matthews correlation coefficient (MCC). ModuleDigger uses Clover [23] to scan the observed and background sequences and enumerates all frequent co-occurrence motifs, finally ranking the finding by a gene-set specificity score. CPModule scans the sequences using a library of PWMs and then uses constraint programming for item set mining approach to enumerate all motif patterns.

For comparing COPS with these programs, we used all three tools to analyze subsets of sequences from the Twi and Pros dataset and from all non-coding regions in the *Drosophila* genome (background). The size of the sequence subsets ranged from 50 to 300 sequences. The number of times a program could identify the motif pairs Bap/Twi, Tin/Twi in the Twi dataset and the motif pairs Ase/Pros, Snail/Pros in the Pros dataset was considered as True Positive count (TP). The False Positive count (FP) corresponds to the number of times these motif pairs were detected in the background. The Matthews Correlation Coefficient (MCC) was calculated as follows:
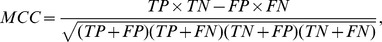
where TN = true negative and FN = false negative.

ModuleDigger and CPModule were accessed at (http://homes.esat.kuleuven.be/~kmarchal/Supplementary_Information_Sun_2009_ModuleDigger/Index.html) and (http://homes.esat.kuleuven.be/~kmarchal/Supplementary_Information_Sun_2011/Index.html) respectively.

### Implementation and Availability of COPS

COPS is implemented in Python (which is platform independent) and can be used directly after download and installation of the SciPy package. We exemplarily cite the memory requirements and data processing time for some of the datasets described in the manuscript.

*Drosophila melanogaster* Twist dataset (scanned for 75 motifs): 484 MB, 158.978 sec.

*Drosophila melanogaster* Pros dataset (scanned for 75 motifs): 552 MB 216.774 sec.

*Mus musculus* Mef2 dataset (scanned for 49 motifs): 2.1GB 283.928 sec.

The main memory consumption is due to the calculation of the score distribution of the PWMs. Therefore longer motifs (i.e. mouse TFs) increase memory usage.

## Results

### COPS: A Computational Tool Detecting TF Motif co-occurrences in Genome-Wide Datasets

**Figure 1:**
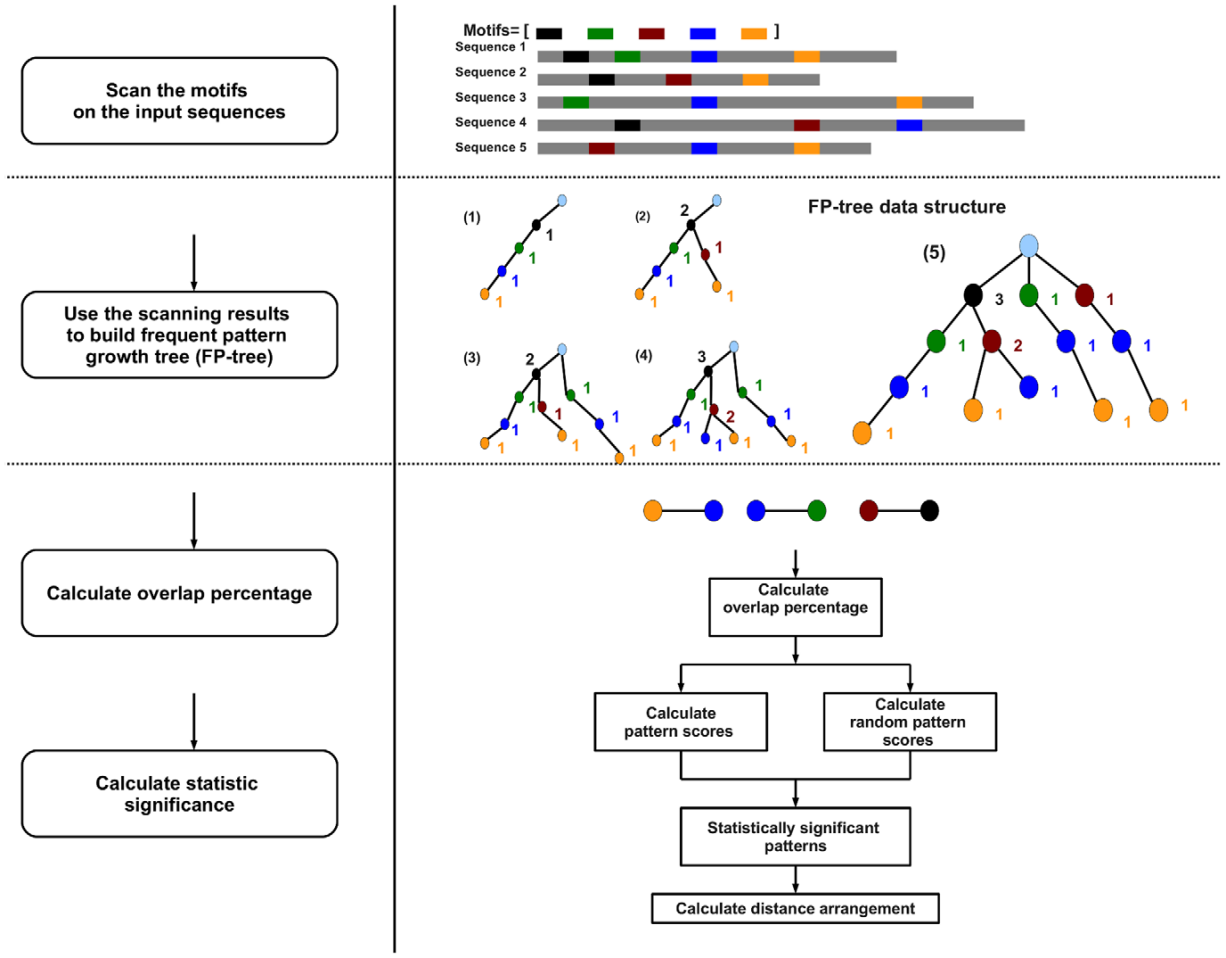
COPS flowchart. COPS first scans the input sequences using all known TF binding motifs annotated in open source databases or retrieved from other resources and builds the frequent pattern (FP)-tree. The statistical significance (Z score) of the motif co-occurrences is calculated by comparing the *log* likelihood score of the frequent pattern to the *log* likelihood score distribution of the background. The percentage of overlap between the motifs of the pair is also calculated and reported. Additionally, COPS offers the option to calculate the preferred distance between co-occurring TF binding motifs and its statistical significance (Z score).

In this study we present COPS (Figure 1), a computational tool that detects statistically significant TFBS co-occurrences in genome-wide datasets consisting of genomic regions bound by a single TF (referred to as “main TF”) *in vivo*, as shown by ChIP-on-Chip, ChIP-Seq or DamID experiments. An analysis with COPS aims at identifying candidate co-regulators of the main TF involved in the control of cell type-specific processes.

Initially, COPS scans the input sequences using all TF binding motifs annotated in open source databases (JASPAR [10], TRANSFAC [11]) or retrieved from other resources (i.e. published optimized or alternative PWMs, Figure S1), thereby building the FP-tree. The FP-tree based approach efficiently compresses large datasets into a condensed data structure and in contrast to the Apriori-like set-generation-and-test method [24] it avoids costly candidate generation and repeated database scans [19], [25]. Therefore employing an FP-tree algorithm makes COPS efficient with respect to both data structure and implementation speed, in particular when mining large datasets. The frequent patterns containing BSs for the main TF are selected for further validation. Each motif co-occurrence pattern is statistically validated based on the *log* likelihood score calculated using two Markov models. The models are trained separately using the adequate input sequences and background sequences assembled from all the non-coding sequences of the respective genomes. The statistical significance cut-off score for motif co-occurrence (Z score) is calculated by comparing the *log* likelihood score of the frequent pattern to the *log* likelihood score distribution of the background. An additional feature implemented in COPS is the calculation of the preferred distances between co-occurring DNA motifs and its statistical significance.

COPS ultimately generates a list of co-occurring TFBS pairs and reports for each of them the statistical significance of the co-occurrence (Z score) and the percentage of overlap between the BSs of the TF pair. Highly overlapping motifs can generate ambiguous results and therefore COPS users may decide to discard such pairs from the list of results. However, before a pair is discarded, users need to consider that TFs of the same family very often bind to highly similar sequences, therefore the properties of the TFs of the pair should be examined. Furthermore, COPS calculates the preferred distance arrangement between the motifs of the detected TF pairs and its statistical significance (p-value). As the requirements of individual users may vary when they employ COPS for data analysis, the parameters provided by the program for each motif pair can be considered accordingly. Generally, a high Z score and a low BS overlap are good indicators of true positive results. Nevertheless, motif pairs are likely to be ranked with a rather low statistical significance score in cases when the reported PWMs fail to represent the actual binding profile of the respective TFs or they are poorly annotated. Therefore, additional parameters defined by the users are sometimes required for further analysis of the results. For instance, the users can resort to databases in order to address whether the co-occurring TFs are expressed in the same tissues as the main TF or whether they are suggested to physically interact with the main TF. Examples of additional means employed for result validation are given in the following sections.

### Analysis of Genomic Regions Bound by the Mesoderm Specification TF Twist in Stage 10 to 11 *Drosophila* Embryos

In order to evaluate the biological relevance of COPS predictions, we analyzed three published genome-wide datasets containing CRMs interacting with several co-regulatory TFs. The first dataset contains the *in vivo* binding regions for five *Drosophila* mesoderm specification TFs, namely Twist (Twi), Myocyte enhancer factor 2 (Mef2), Tinman (Tin), Bagpipe (Bap) and Biniou (Bin), identified by ChIP-on-Chip in stage 10 to 11 embryos [6]. From this dataset, we first analyzed the genomic regions bound by Twi, the master regulator of mesoderm differentiation [26]. At the chosen developmental stages, many of the identified Twi CRMs are bound *in vivo* by all the other mesoderm specifying TFs mentioned above [6]. Therefore, co-occurrences of Twi and Bap/Bin/Mef2/Tin BSs on the selected genomic regions can be considered as “true-positive” results.

**Figure 2:**
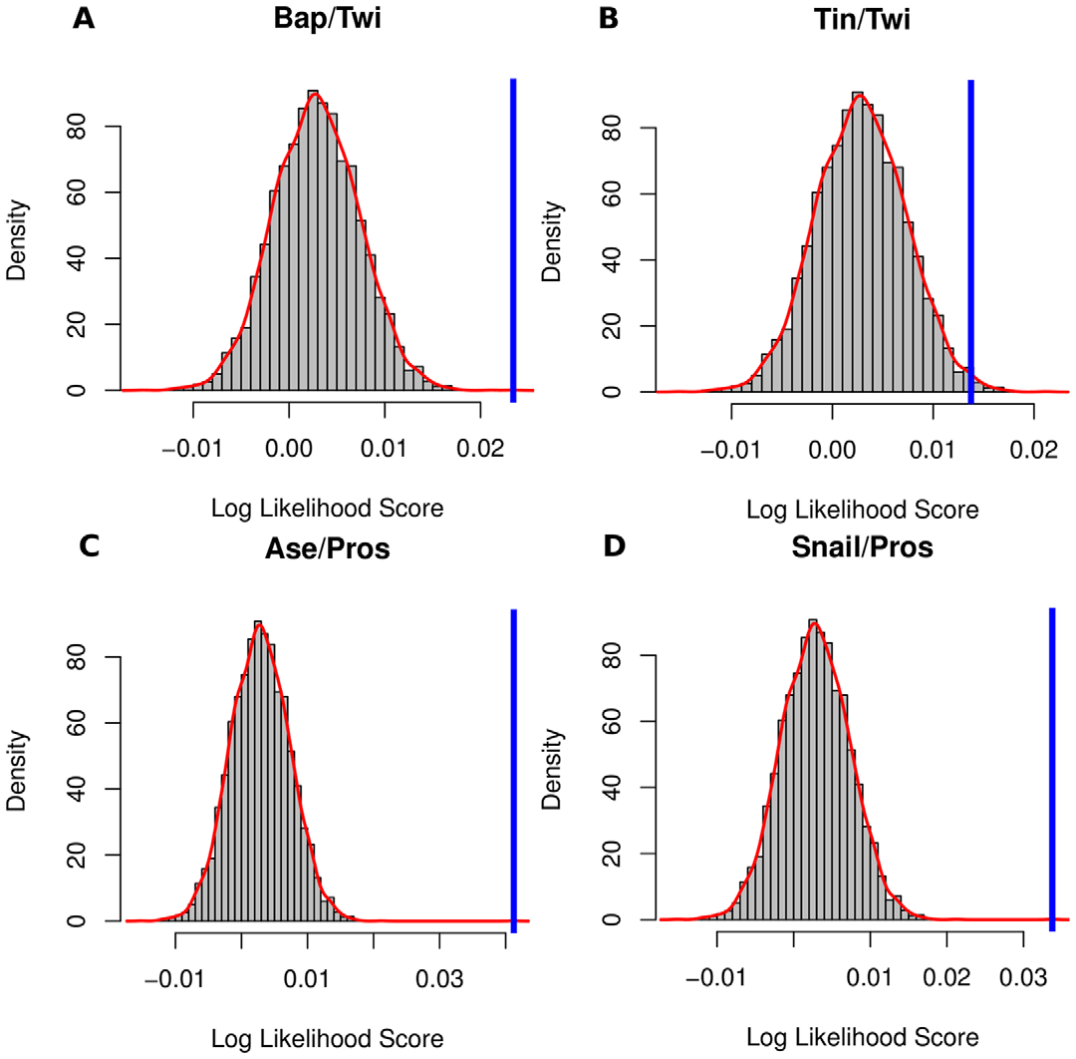
Selected examples of the statistical significance of the identified co-occurrence patterns. The statistical significance of selected motif pairs from the different genome-wide datasets is depicted. The *log* likelihood score (blue bar) of the motif pairs Bap/Twi (A), Tin/Twi (B), Ase/Pros (C) and Snail/Pros (D) is shown in relation to the *log* likelihood score distribution of the background (red curve).

**Figure 3:**
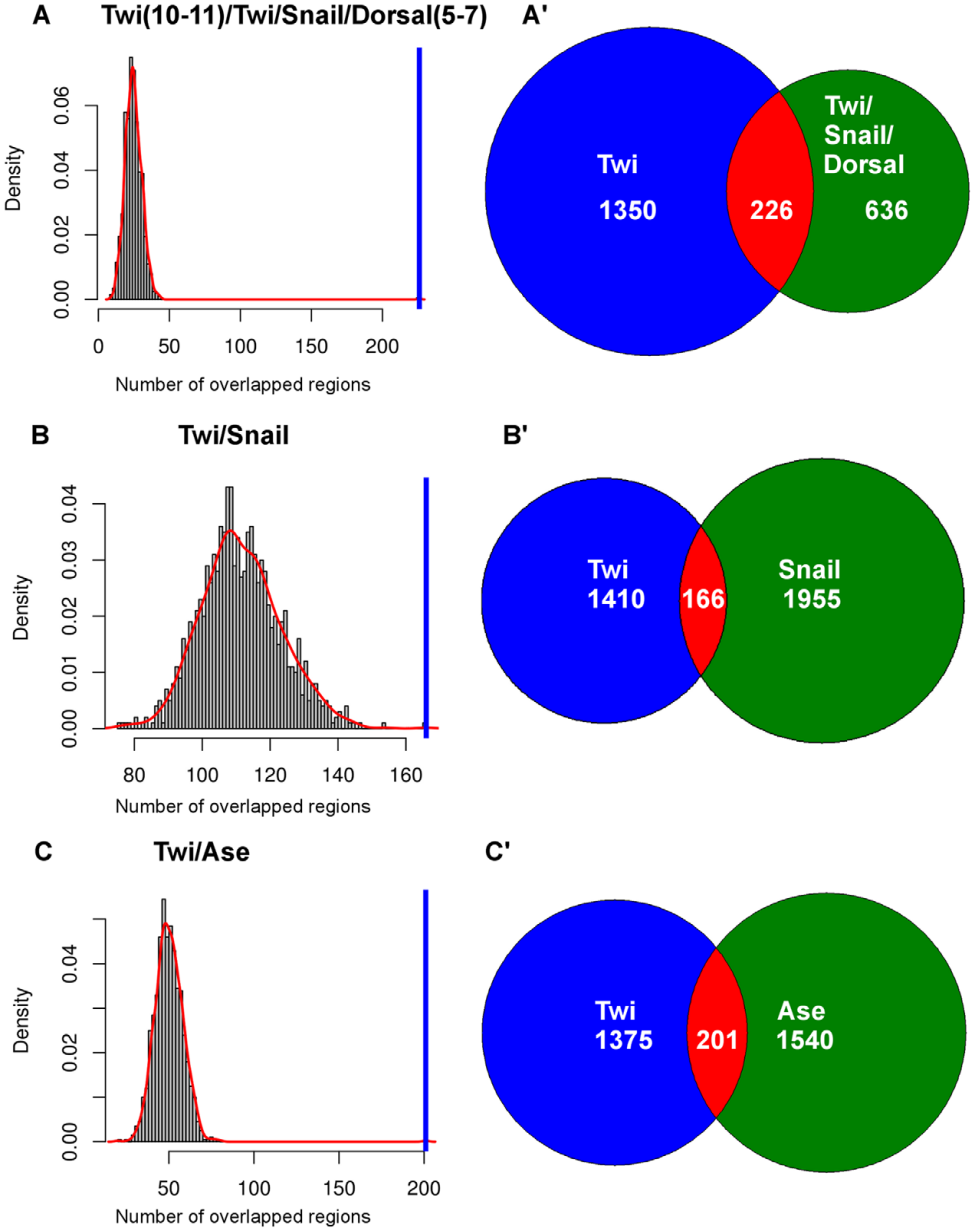
Observed overlap of the genomic regions bound by the TF Twi and its co-regulators. A-C: The observed overlap (blue bar) between genomic regions bound by Twi and genomic regions bound by its known co-regulator Snail and its candidate co-regulator Ase is depicted in comparison to the expected random overlap (red curve). The overlap of genomic regions bound by Twi in stage 10 to 11 [6] with the genomic regions bound by Twi/Snail/Dorsal in stage 5–7 [28] (A), Snail (stage 10 to 11 [7]) (B) and Ase (stage 10 to 11 [7]) (C) is shown. A’-C’: Distribution and overlap (red) of Twi- and Twi/Snail/Dorsal- (A’), Twi- and Snail- (B’) and Twi- and Ase- (C’), bound genomic regions. The regions bound by Twi are shown in blue, the regions bound by the co-regulatory TFs in green and the overlapping regions bound by both Twi and the co-regulatory TFs in red.

Scanning of Twi-interacting regions using all annotated PWMs as well as a number of optimized PWMs described in the literature [6], [12] identified 25 co-occurring TFBSs (statistical significance cut-off: Z score >2.0), including three out of four known Twi co-regulators, Bap, Bin and Tin (Table 1; Table S1). The Twi co-regulator Mef2 could not be retrieved, most likely due to the high -AT- content of the motif. The Z scores (statistical significance) of the frequent patterns Bap/Twi (Figure 2A) and Tin/Twi (Figure 2B) are shown as representative examples. Furthermore, COPS detected BSs for Snail, a TF that together with Twi is involved in the regulation of mesodermal and dorsoventral-patterning genes in early *Drosophila* embryos [27], [28]. In order to address whether the Twi/Snail BS co-occurrences identified by COPS correlate with *in vivo* binding, we used genomic regions shown by ChIP-on-Chip to be combinatorially bound by Twi/Dorsal/Snail *in vivo* in stage 5 to 7 *Drosophila* embryos [28]. Despite the differences in the developmental stages used in the two studies, a comparison of genome-wide Twi binding regions (stage 10 to 11) with Twi/Dorsal/Snail binding regions (stage 5 to 7) revealed a substantial and statistically significant overlap (Table 2, Figure 3A), indicating that Snail interacts with the respective CRMs in consecutive developmental stages and represents a Twi co-regulator in mesoderm specification.

**Table 1:** Classification of TFs co-occurring with Twi on Twi-bound genomic regions in stage 10 to 11 *Drosophila* embryos.

	No. of TFs
Known Twi co-regulators (mesoderm development): Bap, Bin, Tin [6]	3
Known Twi co-regulator in dorsoventral patterning: Snail[28]	1
TFs involved in mesoderm specification/muscle development: Mad, Med, Odd, Usp, Byn	5
TFs with functions in other tissues or with uncharacterized functions	16
**Total Number of TFs**	**25**
% of known Twi co-regulators	16%
% of mesoderm/muscle related TFs	20%

The TFs were classified based on their known co-regulatory function with Twi and their involvement in mesoderm specification and muscle development or other developmental processes.

In addition to the four known Twi co-regulators, we detected co-occurring BSs for five TFs described either to be expressed in mesoderm-derived tissues or to be implicated in muscle development (Table 1; Table S1). Thus, we assumed these factors to function as so-far unknown Twi transcriptional co-regulators in mesoderm development. In sum, 16% of the TFs predicted to bind on Twi-regulated CRMs are known to combinatorially regulate gene expression together with Twi. Moreover, 20% of the predicted TFs control mesoderm-derived tissue development, showing that COPS successfully identified biologically meaningful TF co-occurrences (36% of the detected TF pairs) on Twi CRMs.

Interestingly, COPS detected Twi BSs in combination with BSs for TFs related to nervous system development, among which Deadpan (Dpn), Asense (Ase) and Snail (Table S1). The substantial and statistically significant overlap of genome-wide Twi [6] and Dpn-, Ase- or Snail- [7] binding regions (Figure 3B and 3C, Table 2) suggests *in vivo* implications for the co-occurrence of Twi with neuronal TFs. Since Twi, in addition to its prominent role in mesoderm specification, has been suggested to be important for patterning the neurogenic ectoderm during early embryonic development [29], this result underlines the power of COPS in identifying potential tissue-specific TF co-regulators and uncovering new transcriptional networks regulating specific biological processes.

**Table 2:** Overlap of genomic regions bound by Twi and Twi co-regulatory TFs *in vivo.*

TF Pair	Twi-bound regions	Co-TF bound regions	Observed overlap	Expected overlap	P-value
Twi (st. 10–11)/Twi/Snail/Dorsal (st.5–7)	1576	861	226	24.93	0
Twi/Snail	1576	2121	166	111.60	3.6×10^−6^
Twi/Dpn	1576	1083	92	30.88	0
Twi/Ase	1576	1741	201	50.27	0

Overlap of Twi-bound regions with the genomic regions bound by Twi/Snail/Dorsal in stage 5 to 7 embryos and with the regions bound by the neurogenic TFs Snail, Ase and Dpn in stage 10–11 embryos.

In addition to the Twi-bound genomic regions, we independently analyzed the genomic regions bound by Bin, Tin, Bap and Mef2. In all four sequence-sets, COPS detected co-occurrence of the motif of the respective TF with the motifs of other mesoderm specification factors (Table S1), i.e. Twi/Bin, Tin/Bin and Bap/Bin motif pairs were found on Bin-bound regions.

### Analysis of Genomic Binding Regions for the Neuroblast Differentiation TF Prospero in Stage 10 to 11 *Drosophila* Embryos

The second genome-wide dataset consists of genomic regions bound by the neural stem cell TFs, Prospero (Pros), Ase, Dpn and Snail, as defined by DamID in stage 10 to 11 *Drosophila* embryos [7]. Within the genomic regions bound by the key neuronal stem cell differentiation TF Pros [30], COPS identified 16 co-occurring TFBSs (statistical significance cut-off: Z score >2.0) (Table S2), among which the BSs for the three known Pros co-regulators Snail, Ase and Dpn as well as BSs for seven TFs implicated in neuronal differentiation and nervous system development (information retrieved from FlyBase) (Table 3, Table S2). The statistical significance (Z score) of the frequent patterns Ase/Pros (Figure 2C) and Snail/Pros (Figure 2D) are shown as representative examples. In line with the prominent role of Pros in regulating gene expression in glial cells, the known regulator of glia development Tramtrack (Ttk) was identified as a co-occurring TF on Pros binding genomic regions.

**Table 3:** Classification of TFs co-occurring with Pros in DamID identified genomic regions in stage 10 to 11 *Drosophila* embryos.

	No. of TFs
Known Pros co-regulators in neuroblasts: Snail, Ase, Dpn [7]	3
TFs involved in nervous system development: Ttk, Brk, Btd, Hkb, Deaf1, Ct, H	7
TFs with functions in other tissues or with uncharacterized functions	6
**Total Number of TFs**	**16**
% of known Pros co-regulators	19%
% of nervous system related TFs	44%

The TFs were classified based on their combinatorial activity with Pros and their involvement in nervous system development and other developmental processes.

The genomic regions bound by Ase, Dpn and Snail were independently analyzed and in all cases COPS detected co-occurrence of the motif of the respective TF with the motifs of all other neural stem cell specification factors (Table S2).

### Analysis of Genomic Binding Regions for Mef2a in HL-1 Mouse Cardiomyocytes

In addition to the two *Drosophila* datasets, we analyzed the genome-wide data generated by Schlesinger et al. (2011) using mouse HL-1 cardiomyocytes. In this study [8], ChIP-on-Chip was performed for four key cardiac TFs, Mef2a, GATA-binding protein 4 (GATA4), NK2 TF related, locus 5 (Nkx2.5) and Serum response factor (Srf). Scanning Nkx2.5 genomic binding regions for TFBS co-occurrences (Table 4, Table S3) retrieved BSs of the known Nkx2.5 co-regulator Mef2a. Srf BSs were not detected on Mef2a-bound regions. Two likely explanations are that Srf BSs are only partially represented by TRANSFAC PWMs and that only a small number of Srf binding regions contain Srf BSs [8], [13]. GATA4 BS could not be detected either, which is very likely due to the fact that the GATA1 motif was used for performing the analysis (the GATA4 motif is not available). In line with the prominent role of Nkx2.5 in heart morphogenesis, BSs for three TFs regulating heart development (information retrieved from UniProtKB) were found together with Nkx2.5 BSs (Table 4, Table S3).

**Table 4:** Classification of TFs co-occurring with Nkx2.5 in ChIP-on-Chip identified genomic regions in HL-1 cells.

	No. of TFs
Known Nkx2.5 co-regulator in heart development: Mef2a [8]	1
TFs involved muscle or cardiac development: NFATC2, Sox17 and Prrx2	3
TFs with functions in other tissues or with uncharacterized functions	11
**Total Number of TFs**	**15**
% of known co-regulators	7%
% of potential co-regulators of Nkx2.5 in heart or muscle development	20%

The TFs were classified based on their known interactions with Nkx2.5 and their involvement in muscle or heart development.

The genomic regions bound by Mef2a were independently analyzed and COPS detected co-occurrence of the Mef2a motif with the BS of the co-regulator Nkx2.5 (Table S3). SRF and GATA4 bound genomic regions were not analyzed due to the above mentioned limitations concerning the motifs of these TFs.

### Detection of Short Distance Arrangements between Motif Pairs

Systematic analyses of TFBS arrangements on developmental enhancers have highlighted that random distributions of TFBSs on CRMs are not sufficient for precise regulation of target gene expression [31], [32]. On the other hand, short distance arrangements between TFBSs on CRMs (Δbp<100 bp) have been proposed to mediate protein-protein contacts as well as interactions with co-activators/co-repressors that ultimately result in the efficient formation of higher-order regulatory complexes for precise gene regulation [3], [33]. Since fixed spacing of TFBSs is an integral aspect of transcriptional synergy, COPS was designed to detect statistically significant short distance motif arrangements. After detection of frequent motif pairs, COPS can be employed to search for preferred spatial arrangements between the TFBSs. Here we used COPS to scan for preferred distance arrangements ranging from -1 to 100 bp, using a 10 bp window. Nonetheless COPS presents no limitations concerning the minimum and maximum TFBS distance or the interval (number of bp) used for a TFBS arrangement analysis, since these parameters can be individually specified before scanning the dataset of interest. In the datasets analyzed in this work, COPS reported preferred distance arrangements for several motif pairs (for the full list of results from the individual datasets refer to Tables S1, S2 and S3). Interestingly, COPS detected short distance spacing (<20 bp) for the BSs of the known co-regulatory TF pairs Bap/Twi, Tin/Twi, Bap/Tin, Bin/Tin, Bap/Bin, Pros/Ase, Snail/Twi, Dorsal/Snail and Ase/Dpn as well as for the BSs of potential new co-regulators identified in this study (Table 5, Tables S1, S2 and S3).

**Table 5:** Short distance arrangements between BS of known co-regulatory TFs.

Datasetanalyzed	TF Pair	Preferred Distance Arrangement	p-value
Twi DatasetBap Dataset	Bap/Twi	(−1–9)(9–19)	2.31E-024.34E-02
Tin Dataset	Tin/Twi	(9–19)	3.33E-03
Tin Dataset	Bap/Tin	(9–19)	3.62E-02
Bin DatasetBin Dataset	Bin/Tin	(−1–9)(9–19)	1.32E-022.84E-02
Bin Dataset	Bap/Bin	(−1–9)	3.99E-02
Pros Dataset	Pros/Ase	(−1–9)	3.60E-02
Snail Dataset	Snail/Twi	(9–19)	1.84E-02
Snail DatasetSnail Dataset	Dorsal/Snail	(9–19)(−1–9)	3.64E-023.18E-02
Ase DatasetAse DatasetDpn Dataset	Ase/Dpn	(9–19)(−1–9)(−1–9)	2.01E-021.51E-024.57E-02

The table lists the preferred short distance arrangements (Δbp) between the BSs of known co-regulatory TF pairs, as reported by COPS by analyzing different genome-wide datasets (first column). The last column shows the statistical significance (p-value) of the short distance arrangement for each pair of TFBSs. Only pairs with a motif spacing of <20 bp are listed in the table.

Next, we decided to address the biological significance of the close spacing of TFBSs detected by the software using the TF pairs of the known co-regulators Bin/Twi, Ase/Dpn and the novel co-regulatory pair Mad/Twi detected by COPS in this study. All selected pairs showed a preferred close distance motif spacing of -1 to 9 bp. Our approach involved scanning all non-coding regions of the *Drosophila* genome for sequences containing the above mentioned TFBS pairs at a distance of -1–9 bp as reported by COPS and examining the GO terms of the genes associated with these regions. In all three cases, genes associated with these specific short-distance TF combinations exhibited similar functional annotations as the respective TF pair (Table S4). In particular, among the genes associated with the Ase/Dpn short-distance TF pair we found enrichment of GO terms related to cell division (i.e. “mitotic cell cycle”, “regulation of mitotic cell cycle”, “positive regulation of S phase of mitotic cell cycle”) (Table S4), in line with the role of Ase and Dpn in neural stem cell division [7]. Similarly, genes coupled to the Bin/Twi short-distance TF pair displayed the following GO terms enriched, “visceral muscle development”, “larval visceral muscle development” and “somatic muscle development” (Table S4), in agreement with the role of Twi as a master regulator of mesoderm development [26] and of Bin as the main coordinator of visceral muscle differentiation [34]. Interestingly, genes associated with regions containing closely spaced Mad/Twi BSs were found enriched for the GO terms “cardioblast differentiation”, “heart development” and “pericardial cell differentiation” (Table S4). As Mad, Twi and Tin have been shown to combinatorially act on selected cardioblast enhancers [35], our results raise the possibility that their combined activity might be of broader relevance for the regulation of enhancers controlling cardiac identity. Therefore, preferred close distance arrangements detected by COPS can be considered as evidence of transcriptional synergy.

### Comparison of COPS with Other Computational Approaches

The advantage of the multiple sequence tool COPS, in comparison to the single sequence tools Cister [36] and ClusterBuster [37] is its more accurate prediction of TFBS combinations present in CRMs associated with gene sets displaying similar tissue-specific expression. Even though the above-mentioned programs are very efficient in detecting motif combinations on single sequences, they cannot retrieve information concerning groups of genes regulated by enhancers with similar architecture in terms of motif composition.

**Figure 4:**
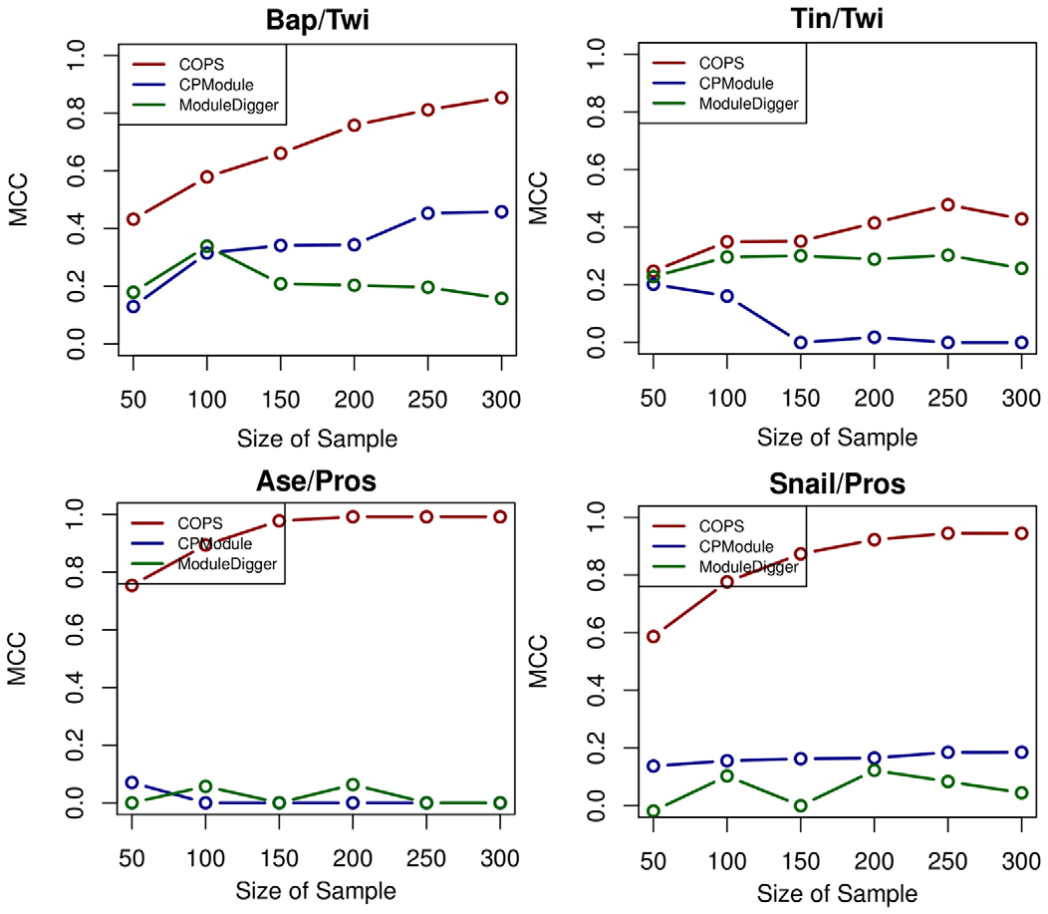
Comparison of COPS to two other computational approaches. The plots depict the Matthews Correlation Coefficient (MCC) values as determined using COPS and the published tools CPModule and ModuleDigger, for the co-occurrence patterns Bap/Twi, Tin/Twi, Ase/Pros and Snail/Pros in sequence-sets of increasing size (ranging from 50 to 300 sequences). The MCC values used for evaluating the performance of the different computational tools were calculated as described in the Materials and Methods. The red line illustrates the performance of COPS, the blue line CPModule and the green line ModuleDigger.

The performance of COPS was directly compared to available multiple sequence tools, namely Compo [38], ModuleDigger [21] and CPModule [22]. Our attempt to use Compo for analyzing the datasets scanned with COPS was not successful, since the software was not able to handle such large numbers of sequences (in some cases >2000 sequences i.e. Pros dataset) and extensive motif collections (>70 motifs). Therefore we did not proceed with a direct comparison of the performance of the two programs. The comparison of COPS to ModuleDigger [21] and CPModule [22] was carried out using sequence-sets of increasing size from the Twi and Pros datasets and the motif pairs Twi/Bin, Bap/Twi, Tin/Twi, and Ase/Pros, Snail/Pros respectively (described in detail in Materials and Methods). As shown in Figure 4, the performance of COPS with respect to the MCC value is comparable or superior to CPModule at differently sized sequence sets with different TF pairs, while COPS outperforms ModuleDigger in every run.

## Discussion

Genome-wide analyses of TF-DNA interactions ultimately aim at unravelling the molecular mechanisms underlying the control of gene expression. As it has been established that the combinatorial input of different TFs on CRMs is a key determinant of spatiotemporal regulation of gene expression, detection of TFBS co-occurrences provides an excellent starting point for identifying TF combinations with tissue-specific transcriptional outputs. To this end, we have developed COPS, a new computational tool which scans genomic sequences for statistically significant TF motif co-occurrences. The performance of COPS with respect to the biological significance of the detected co-occurring TFBS pairs was evaluated by analyzing three independent genome-wide datasets. In all cases, COPS successfully detected co-occurrence of each TF with its known co-regulators. As genome-wide data are available for several of these co-regulators, we could show that co-occurrence of these motif pairs indeed correlates with *in vivo* combinatorial binding. Furthermore, for all analyzed TFs COPS reported co-occurrences with TFs involved in common tissue-specific processes. Therefore COPS is applicable for identifying potential transcriptional co-regulators of a TF of interest.

One advantage of COPS is the use of a FP-tree based data mining approach [19], which avoids the costly candidate generation and testing and is therefore time-efficient, especially when mining large datasets. Moreover, the calculation of the statistical significance of the frequent motif patterns is more dependent on DNA sequence content and to a lesser extent on sequence length, thus eliminating the requirement for normalization of the motif frequencies against the sequence length. Importantly, COPS is capable of efficiently scanning large datasets (i.e. the Snail DamID dataset consisting of 4000 sequences) for extensive motif collections. Overall, the above-mentioned parameters render COPS a powerful, time-efficient and statistically reliable computational tool.

Notably, COPS is not restricted to the detection of motif pairs, but it can also be used for identifying longer co-occurrence patterns, namely combinations of three or more TFBSs. However, the number of motif combinations dramatically expands when scanning for longer patterns using large motif collections, hence resulting in increased memory requirements and processing time. We exemplarily mention that when a *Drosophila* genome-wide dataset is scanned for detecting combinations of three TFBSs using the whole collection of *Drosophila* TF motifs (75 motifs) COPS will have to scan the sequences for a total number of 67525 frequent patterns. Therefore when possible, we advice COPS users to preselect a smaller subset of motifs in analyses involving longer patterns, in order to facilitate their analysis.

One feature of COPS not found in other sequence-screening tools is the detection of distance preferences between co-occurring motifs. The defined spatial organization of TFBSs on CRMs is critical for the proper assembly of functional regulatory complexes, since protein-protein interactions very often depend on favourable arrangements of BSs [3], [5], [33], [39], [40], [41]. Protein complex formation between TFs binding at adjacent BSs can explain how TFs with degenerate DNA-binding specificity precisely regulate their target genes in a cell type-specific manner, either by modifying their DNA recognition properties [42] or by exhibiting a synergistic activity [3], [41], [43]. Therefore, preferred close distance arrangements of TFBSs reported by COPS raise the possibility of direct interactions between the respective TFs. As we showed in this study, genomic regions containing closely spaced TFBS pairs (as reported by COPS) are associated to similar gene classes that reflect the properties of the TFs of the pair, therefore close distance arrangement of TF motifs may at least in some cases indicate cell-type specific combinatorial activity of the respective TFs.

When analyzing sequences with bioinformatics tools such as COPS, a parameter that should be taken into consideration is the important role of PWMs. TF co-occurrences are likely to be falsely omitted if the reported PWMs fail to represent the actual binding profile of the respective TFs or if they are poorly annotated. Furthermore, false positive results may be obtained due to degenerate PWMs that are frequently encountered in the genome and are therefore likely to be part of co-occurrence patterns on multiple genomic regions. An additional critical parameter is the definition of the genomic regions that will be considered as “TF-bound DNA regions”. In contrast to the regions defined by ChIP-Seq, which are usually in the range of a few hundreds base-pairs long, DamID- and ChIP-on-Chip- detected regions often extend up to several kilo base-pairs. In such cases, the region that is bound by the TF *in vivo* is not always easy to detect due to a decrease of the signal to noise ratio. Moreover, similarly to all computational routines, COPS cannot capture several aspects of *in vivo* transcriptional regulation. First of all, recruitment of TFs can occur without the presence of recognizable TF motifs on the respective genomic region. In such cases, genomic localization of the TF is mediated via binding at distal sites followed by DNA looping or via protein-protein interactions [44]. For instance, when Mef2a- and Nkx2.5-bound sequences were analyzed in our study, COPS failed to detect the BSs of the known co-regulator Srf due to the fact that Srf binding at target sequences largely relies on protein-protein interactions and to a lesser extent on the recognition of consensus sequences [8], [44]. In addition, as epigenetic modifications often define the accessibility of genomic regions to TFs, TFBSs detected by COPS might not be occupied *in vivo* [44], [45], [46]. Therefore, genome-wide data on histone modifications could be used to optimize the interpretation of results obtained by COPS. Finally, detection of co-occurring BSs by COPS does not necessarily mean that the respective TFs combinatorially interact with the CRMs, but they could also be occupied by the TFs in different tissues or at different developmental stages.

In sum, COPS is a potent computational tool applicable for identifying potential transcriptional co-regulators that define context-dependent transcriptional outputs. In combination with genome-wide data for TF-DNA interactions, histone modifications and protein-protein interactions COPS allows the elucidation of cell type-specific regulatory networks.

## Supporting Information

Figure S1 Motif logos for the main TFs analyzed in the study. The logos of the motifs used for scanning for BSs of the main TFs from all three datasets are depicted in this figure. All other motif logos can be found in the open source databases TRANSFAC and JASPAR.
(TIF)

Table S1 Analysis of genomic regions bound by mesoderm specification TFs in stage 10 to 11 *Drosophila* embryos. The table includes all results generated by analyzing Twi-, Tin-, Mef2-, Bin- and Bap-bound genomic regions using COPS. The TF motif pairs found to co-occur are listed in column A. Known co-regulators are shown in bold. Candidate co-regulatory TFs expressed in the muscle/mesoderm are highlighted in yellow. Column B shows the statistical significance of the co-occurrence pattern (Z score) and column C the percentage of motif overlap as calculated by the program. The tissue-specific expression of the co-occurring TF of each pair as reported in FlyBase is shown in column D and the literature supporting a co-regulatory role with the main TF in column E. Columns G-M show the preferred distance arrangements between the TF motifs of the different pairs and their statistical significance.
(XLS)

Table S2 Analysis of genomic regions bound by neuroblast differentiation TFs in stage 10 to 11 *Drosophila* embryos. The table includes all the results generated by analyzing Pros-, Ase, Dpn- and Snail-bound genomic regions using COPS. The TF motif pairs found to co-occur are listed in column A. Known co-regulators are shown in bold. Candidate co-regulatory TFs expressed in neuroblasts/nervous system are highlighted in light blue. Column B shows the statistical significance of the co-occurrence pattern (Z score) and column C the percentage of motif overlap as calculated by the program. The tissue-specific expression of the co-occurring TF of each pair as reported in FlyBase is shown in column D and the literature supporting a co-regulatory role with the main TF in column E. Columns G-O show the preferred distance arrangements between the TF motifs of the different pairs and their statistical significance.
(XLS)

Table S3 Analysis of genomic regions bound by cardiac mesoderm specification TFs in HL-1 mouse cardiomyocytes. The table includes all the results generated by analyzing Nkx2.5- and Mef2a-bound genomic regions using COPS. The TF motif pairs found to co-occur are listed in column A. Known co-regulators are shown in bold. Candidate co-regulatory TFs expressed in the heart are highlighted in orange. Column B shows the statistical significance of the co-occurrence pattern (Z score) and column C the percentage of motif overlap as calculated by the program. The tissue-specific expression and developmental functions of the co-occurring TF of each pair as reported in UniProtKB is shown in column D and the literature supporting a co-regulatory role with the main TF in column E. Columns G-K show the preferred distance arrangements between the TF motifs of the different pairs and their statistical significance.
(XLS)

Table S4 Overrepresented GO terms of genes associated to genomic regions showing close distance arrangements of TFBS pairs. The table shows the GO terms (biological process, Column B) that were found overrepresented among the genes associated with genomic regions containing the motif pairs Bin/Twi, Mad/Twi and Ase/Dpn (Column A of each worksheet) at a close distance arrangement of −1–9 bp. The P value for each overrepresented GO term is shown in Column G. The P value was calculated by comparing the number of genes linked to each GO term to the total number of genes associated with genomic regions containing the short distance motif pair (Column C vs Column D) in relation to the number of genes linked to the GO term and associated to regions without the short distance motif pair (Column E vs Column F).
(XLS)
