# Supplementary figures and images for: COPS: Detecting Co-Occurrence and Spatial Arrangement of Transcription Factor Binding Motifs in Genome-Wide Datasets
Source: PLoS One. 2012 Dec 18;7(12):e52055. doi: 10.1371/journal.pone.0052055 (PMC3525548; doi:10.1371/journal.pone.0052055)

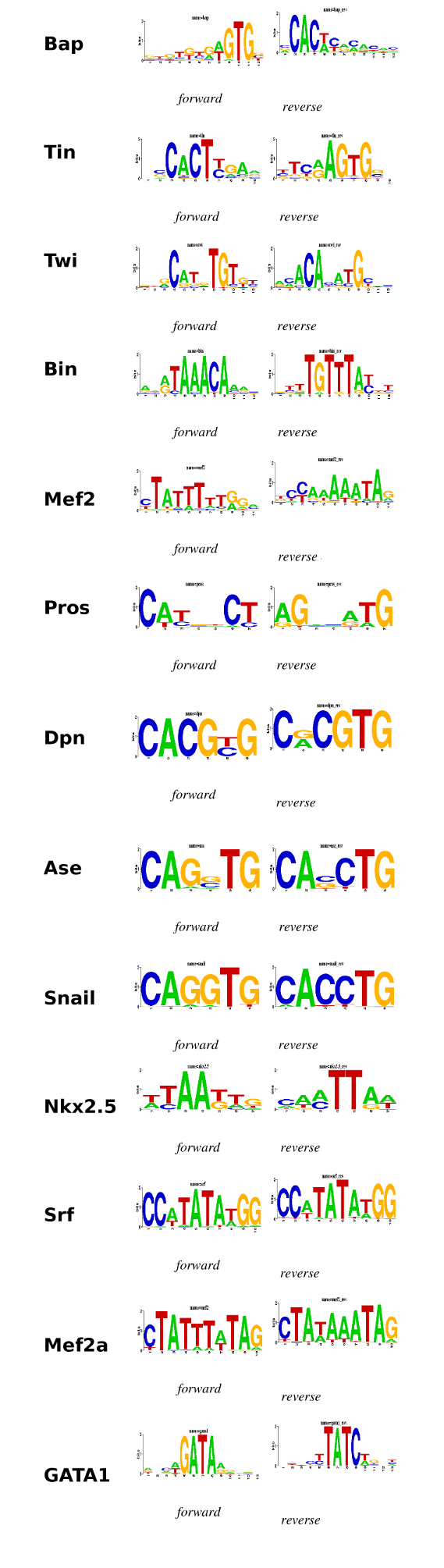

Supplement: Figure S1 — Motif logos for the main TFs analyzed in the study. The logos of the motifs used for scanning for BSs of the main TFs from all three datasets are depicted in this figure. All other motif logos can be found in the open source databases TRANSFAC and JASPAR. (TIF) [file pone.0052055.s001.tif]
